# A Complex Role for FGF-2 in Self-Renewal, Survival, and Adhesion of Human Embryonic Stem Cells

**DOI:** 10.1002/stem.128

**Published:** 2009-08

**Authors:** Livia Eiselleova, Kamil Matulka, Vitezslav Kriz, Michaela Kunova, Zuzana Schmidtova, Jakub Neradil, Boris Tichy, Dana Dvorakova, Sarka Pospisilova, Ales Hampl, Petr Dvorak

**Affiliations:** a Department of Biology, Faculty of Medicine University Hospital Brno and Faculty of Medicine, Masaryk UniversityBrno, Czech Republic; b Center for Chemical Genetics, Faculty of Medicine, and University Hospital Brno and Faculty of Medicine, Masaryk UniversityBrno, Czech Republic; c Department of Internal Medicine–Hemato-oncology, University Hospital Brno and Faculty of Medicine, Masaryk UniversityBrno, Czech Republic; d Department of Molecular Embryology, Institute of Experimental Medicine, Academy of Sciences of the Czech RepublicPrague, Czech Republic; e Center for Cell Therapy and Tissue Repair, 2nd Faculty of Medicine, Charles UniversityPrague, Czech Republic

**Keywords:** Fibroblast growth factor-2, Human ESCs, Self-renewal, Cell survival, Adhesion

## Abstract

The transcription program that is responsible for the pluripotency of human ESCs (hESCs) is believed to be comaintained by exogenous fibroblast growth factor-2 (FGF-2), which activates FGF receptors (FGFRs) and stimulates the mitogen-activated protein kinase (MAPK) pathway. However, the same pathway is stimulated by insulin receptors, insulin-like growth factor 1 receptors, and epidermal growth factor receptors. This mechanism is further complicated by intracrine FGF signals. Thus, the molecular mechanisms by which FGF-2 promotes the undifferentiated growth of hESCs are unclear. Here we show that, in undifferentiated hESCs, exogenous FGF-2 stimulated the expression of stem cell genes while suppressing cell death and apoptosis genes. Inhibition of autocrine FGF signaling caused upregulation of differentiation-related genes and downregulation of stem cell genes. Thus, exogenous FGF-2 reinforced the pluripotency maintenance program of intracrine FGF-2 signaling. Consistent with this hypothesis, expression of endogenous FGF-2 decreased during hESC differentiation and FGF-2 knockdown-induced hESC differentiation. In addition, FGF-2 signaling via FGFR2 activated MAPK kinase/extracellular signal-regulated kinase and AKT kinases, protected hESC from stress-induced cell death, and increased hESC adhesion and cloning efficiency. This stimulation of self-renewal, cell survival, and adhesion by exogenous and endogenous FGF-2 may synergize to maintain the undifferentiated growth of hESCs. Stem Cells *2009;27:1847–1857*

## INTRODUCTION

Human ESCs (hESCs) are potential models of human development and disease and an unlimited source of cell replacement therapies. The crucial challenge for these applications is to understand how to maintain and expand hESCs in an undifferentiated state without acquiring genetic abnormalities. The current optimized cell culture conditions that support long-term undifferentiated growth of hESCs include the extrinsic activities of several growth factors and a supporting feeder cell layer. Feeder-free culture conditions are less supportive for the unlimited undifferentiated culture of hESCs and require feeder cell-conditioned medium or elevated concentrations of growth factors. The most common growth factors supplements for hESC culture media that promote self-renewal are fibroblast growth factor-2 (FGF-2) [1,2], activin A [3–5], transforming growth factor β1 (TGFβ1) [6], and Wnt1 and 3 [7–9]. Although the majority of these growth factors are not necessary for culture of hESCs with feeder cells or feeder cell-conditioned media, FGF-2 is required. This seems to be paradoxical because FGF-2 stimulates trophectoderm differentiation [10], the early stages of endodermal development [11], differentiation of endoderm-derived pancreatic cells [12], and differentiation of mesoderm-derived cardiovascular progenitors [13]. One possible explanation is that FGF-2 has different effects on undifferentiated cells than cells that are already committed to differentiate.

In undifferentiated hESCs FGF-2 likely promotes self-renewal in several ways. It directly activates the mitogen-activated protein kinase (MAPK) pathway [1,14], whereas indirectly it acts on fibroblast feeder cells to modulate TGFβ1 and activin A signaling, which together support hESC self-renewal [15,16]. FGF-2 also induces the production of TGFβ and insulin-like growth factor-II (IGF-II) from hESC-derived fibroblast-like cells that define a self-renewal-supporting hESC niche [17]. More recently, one study has suggested that extrinsic FGF-2 signaling directly regulates *NANOG* promoter activity [18]. Remarkably, although the activation of the MAPK cascade by exogenous FGF-2 stimulates mouse ESC proliferation [19], it does not stimulate hESC proliferation [1,14]. There are at least two possible explanations for this disparity in hESCs. First, the MAPK pathway may be predominantly activated by insulin receptors, insulin-like growth factor 1 receptors (IGF1Rs), and epidermal growth factor receptors (EGFRs) [20] in hESCs, thus buffering the action of exogenous FGF-2 on cell proliferation. Second, intracrine FGF activities in hESCs may maintain high levels of MAPK activation such that proliferation is not further enhanced by extrinsic FGF signals. In support of the second hypothesis, mouse ESCs were suggested to have an innate program for self-renewal that does not require extrinsic signals [21]. The excess of exogenous growth factors may also have receptor-independent mechanisms that negatively regulate pathways that direct pluripotent cell differentiation. Consistent with these proposed mechanisms, FGF-2 is highly expressed in various somatic cell types, where it has established intrinsic function in the regulation of cell proliferation, differentiation, and survival [22,23].

In this study, we suggested that intrinsic FGF-2 signaling maintained the undifferentiated growth and survival of hESCs. In contrast, exogenous FGF-2 had partially overlapping functions in the maintenance of hESC undifferentiated growth and survival, but in addition, stimulated hESC adhesion that indirectly contributed to the maintenance of hESCs pluripotency. Thus, we propose that the maintenance of hESC self-renewal by intracrine FGF-2 is enhanced by extrinsic FGF-2 signals.

## MATERIALS AND METHODS

### Culture of hESCs

Karyotypically normal CCTL12 (46, XX) and CCTL14 (46, XX) hESC lines [24] were routinely maintained in Dulbecco's modified Eagle medium (DMEM)/F12 supplemented with 15% (vol/vol) knockout serum replacement, L-glutamine, MEM nonessential amino acids, 0.5% (vol/vol) penicillin-streptomycin, 5 ng/ml FGF-2 (all media components from Invitrogen, Carlsbad, CA, http://www.invitrogen.com), and β-2 mercaptoethanol (Sigma-Aldrich, St. Louis, http://www.sigmaaldrich.com) on mitotically inactivated embryonic fibroblasts from the CF 1 mouse strain. Passage numbers 21-69 (CCTL12) and 22-57 (CCTL14) were used for all experiments.

### DNA Array Analysis

hESCs were cultured in standard FGF-2 (5 ng/ml)-supplemented medium or in medium without FGF-2 but supplemented with 20 μM SU5402 (Calbiochem, San Diego, http://www.emdbiosciences.com) for 6 days. Control cells for both treatments were cultured in medium without FGF-2. Two independent replicates were hybridized to Agilent Human 1A v2 chips containing 60-mer oligonucleotide probes covering transcripts for approximately 20,000 annotated human genes (Agilent Technologies, Palo Alto, CA, http://www.agilent.com). Genes that were equally expressed in both replicates were selected for further analysis. Functional annotation of genes was performed according to the KEGG pathways using the FatiGOplus program [25].

### Immunoblotting and Immunocytochemistry

For immunoblot analysis of FGF-2, hESCs lysates containing equal amounts of total protein were mixed with 2× Laemmli sample buffer, separated by SDS-PAGE, and electrotransferred onto Hybond P membrane (Amersham Pharmacia Biotech, Buckinghamshire, U.K., http://www.gelifesciences.com). Membranes were incubated with mouse FB-8 monoclonal antibody to FGF-2 (Sigma-Aldrich). Mouse monoclonal antibody to α-tubulin (ExBio, Prague, Czech Republic, http://www.exbio.cz) was used to normalize loading. Membranes were incubated with appropriate horseradish peroxidase-conjugated secondary antibodies, and protein bands were visualized using the chemiluminescence detection reagent ECL+Plus (Amersham).

For in situ detection, hESCs growing on mouse feeder layers were fixed either with 95% ethanol and 1% acetic acid, or 4% paraformaldehyde, blocked with 5% normal goat serum or bovine serum albumin (BSA), and incubated with primary antibodies diluted in blocking solution. Primary antibodies included rabbit polyclonal antibody to FGF-2 (Sigma-Aldrich), mouse monoclonal antibody to Oct4 (Santa Cruz Biotechnology, Santa Cruz, CA, http://www.scbt.com), rabbit polyclonal antibody to Nanog (Santa Cruz Biotechnology), and rabbit polyclonal antibody to Ki-67 (Santa Cruz Biotechnology). Unbound antibody was removed, and cells were incubated with the appropriate secondary antibodies conjugated to peroxidase (Sigma-Aldrich), Alexa Fluor 488 (Invitrogen), and/or Alexa Fluor 594 (Invitrogen). Cell nuclei were stained with 4′,6-diamidino-2-phenylindole (DAPI) and mounted in Mowiol (Polysciences, Warrington, PA, http://www.polysciences.com) containing 1,4-diazobicyclo-[2.2.2.]-octane to prevent fading. Microscopic analysis was performed using an Olympus FluoView 500 laser scanning microscope (Olympus, Tokyo, http://www.olympus-global.com).

### Reverse Transcription-Polymerase Chain Reaction and Quantitative Real-Time Reverse Transcription-Polymerase Chain Reaction Analysis

Reverse transcription-polymerase chain reaction (RT-PCR) for FGF-2 and real-time RT-PCR for FGF receptors (FGFRs) were performed as previously described [1,16]. Supporting information Table 1 lists the primes and probes used for RT-PCR and real-time RT-PCR. Samples from sorted SSEA3-positive cells were kindly provided by P. W. Andrews, University of Sheffield, Sheffield, U.K. Samples of the independent hESC line HS237 were kindly provided by O. Hovatta, Karolinska Institutet, Huddinge, Sweden.

### FGF-2 Binding Assay

Biotinylation of FGF-2 was performed using the EZ-Link Micro NHS-PEO4-Biotinylation kit (Thermo Fisher Scientific, Bonn, Germany, http://www.thermo.com) according to the manufacturer's instructions. For the receptor-binding assay, hESCs were cultured with or without FGF-2 for 5 days. The cells were rinsed extensively and incubated on ice in DMEM/H/B (DMEM/F12, 15 mM HEPES, and 0.5% BSA) containing 10, 50, or 100 ng/ml biotinylated FGF-2 for 30, 60, or 90 minutes. Control cells were maintained in DMEM/H/B. The cells were rinsed three times with DMEM/H/B, fixed in 95% ethanol/1% acetic acid, and quenched with 5% normal goat serum/1% BSA. This was followed by incubation with streptavidin/fluorescein isothiocyanate (Vector Laboratories, Burlingame, CA, http://www.vectorlabs.com). Cells were rinsed, counterstained with DAPI, and mounted in Mowiol containing 1,4-diazobicyclo-[2.2.2.]-octane. Fluorescence signal analysis was performed using an Olympus FluoView 500 laser scanning microscope (Olympus).

### shRNA-Mediated Gene Silencing

The *FGF-2* gene was knocked down using validated small hairpin RNA (shRNA) oligonucleotides: 5′-ACC*GGATTCTGGAGTATACTTATTCAAGAGATAAGTATACTCCAGAATCC*TTTTTC-3′ (sense); 5′-TGCAGAAAAA*GGATTCTGGAGTATACTTA*TCTCTTGAA*TAAGTATACTCCAGAATC*-3′ (antisense) of FGF-2 mRNA (Ambion, Austin, TX, http://www.ambion.com) cloned into the psiSTRIKE-Hygromycin vector (Promega, Madison, WI, http://www.promega.com). Nonsilencing shRNA oligonucleotides 5′-Acc*gcttgaaccgccagatcta*ttcaagaga*tagatctggcggttcaagc*tttttc-3′ (sense) and 5′-tgcagaaaaa*gcttgaaccgccagatcta*tctcttgaa*tagatctggcggttcaag*-3′ (antisense) [26] were cloned in to the same vector to serve as a negative control. Low passage hESCs (passage 31) at 80% confluency growing on hygromycin-resistant mouse feeder cells (Millipore, Billerica, MA, http://www.millipore.com) were transfected using Lipofectamine 2000 (Invitrogen) according to the manufacturer's instructions. Stable integration of the plasmid encoding FGF-2 shRNA was selected using 75 μg/ml hygromycin for 2-3 weeks. Resulting hESCs colonies were tested for the presence of psiSTRIKE-Hygromycin vector by PCR (primers: 5′-GCGATTAAGTTGGGTAAC-3′; 5′-ACGCAATTAATGTGAGTTAG-3′; resulting in a 620-bp fragment), replated onto normal feeder cells, and expanded for further experiments. The knockdown effect for FGF-2 was detected in hESC subclones by immunoblot analysis.

### Phospho-Specific Protein Arrays and Mitogen-Activated Protein Kinase Assay

The relative phosphorylation status of tyrosine kinase receptors was assayed using the Human Phospho-Receptor Tyrosine Kinase array (R&D Systems, Minneapolis, http://www.rndsystems.com) according to the manufacturer's instructions. The phosphorylation of mitogen-activated protein kinase kinase 1 and 2 (MEK1/2) and their substrate extracellular signal-regulated kinases (ERK1/2), was determined by immunoblot analysis. Primary antibodies included rabbit polyclonal antibody to phospho MEK1/2 (New England Biolabs, Ipswich, MA, http://www.neb.com), rabbit polyclonal antibody to MEK1/2 (New England Biolabs), mouse monoclonal antibody to phospho ERK1/2 (Santa Cruz Biotechnology), and rabbit polyclonal antibody to ERK1/2 (Santa Cruz Biotechnology). After incubation with peroxidase-conjugated secondary antibodies (Sigma-Aldrich), protein bands were visualized using the chemiluminescence detection reagent ECL+Plus (Amersham Pharmacia Biotech). Determination of the relative phosphorylation level of MAPKs including serine/threonine kinases was performed using the Human Phospho-MAPK array kit (R&D Systems) according to the manufacturer's instructions.

### Cell Death and Viability Assays

Detection of apoptotic hESCs after ionizing radiation and oxidative stress was performed using the ApopTag Plus Fluorescein *In Situ* Apoptosis detection kit (Millipore) according to the manufacturer's instructions. The percentage of apoptotic cells was determined by fluorescence microscopy with an Olympus FluoView 500 laser scanning microscope (Olympus). Samples of 1,000-2,000 cells growing either in the presence or absence of 18-kDa FGF-2 on 35-mm dishes (ibidi GmbH, Martinsried, Germany, http://www.ibidi.de) were counted for each experimental group.

The cleavage of poly(ADP-ribose) polymerase-1 (PARP-1) and lamin B in hESCs subjected to ionizing radiation and oxidative stress was used to assess the activation of caspase-3 and apoptotic disintegration of the nuclear envelope, respectively. The cleavage products were detected by immunoblotting with the following primary antibodies: rabbit polyclonal antibody to PARP (Santa Cruz Biotechnology) and goat polyclonal antibody to lamin B (Santa Cruz Biotechnology). After incubation with peroxidase-conjugated secondary antibodies (Sigma-Aldrich), protein bands were visualized using the chemiluminescence detection reagent ECL+Plus (Amersham Pharmacia Biotech). Cell viability and cell counts were determined using the ViCell XR (Immunotech a.s., a Beckman Coulter Company, Prague, Czech Republic, http://www.immunotech.cz).

### Cloning Efficiency Assay

Human ESCs were FGF-2 starved for 6 hours, washed with PBS, treated with TrypLE reagent (Invitrogen) for 3 minutes, and gently dissociated to single cells by pipetting either in medium with or without FGF-2 (50-100 ng/ml). Then, TrypLE reagent was washed out by centrifugation, and single cell suspensions of cells were preincubated in fresh medium with or without FGF-2 (50-100 ng/ml) for 1 hour. Dissociated cells were seeded at the density of 1 × 10^4^/cm^2^ on 24-well feeder cell-coated plates and cultured overnight in the same media with or without high concentrations of FGF-2. The following day, FGF-2-free and FGF-2-high culture medium was replaced by a standard culture medium supplemented with 5 ng/ml FGF-2. After 5 days of culture, when hESCs formed colonies that were homogenous in size, the cells were fixed by 4% paraformaldehyde and stained with the Alkaline Phosphatase Detection kit (Millipore). To determine the cloning efficiency, each well of a 24-well plate was photographed, and colony counting was performed using ImageJ software (Rasband WS, U.S. National Institutes of Health, Bethesda, MD, http://rsbweb.nih.gov/ij/).

## RESULTS

### Gene Expression Analysis Highlights Importance of Intrinsic FGF Signals in hESCs

Human ESCs express high levels of endogenous FGF-2 that might signal in autocrine, paracrine, or intracrine ways. To address the effects of intrinsic FGF-2 signaling on gene expression, we blocked autocrine/paracrine FGF signaling with the inhibitor of FGFR kinases SU5402 in cells that were starved of exogenous FGF-2, and changes in the global gene expression pattern were assessed using DNA microarrays. In parallel controls, uninhibited hESCs maintained in medium supplemented with or without FGF-2 were also analyzed using DNA microarrays. In two independent experiments, we observed characteristic gene expression changes; however, the number and magnitude of the fold change of differentially expressed genes were higher in cells exposed to SU5402. Specifically, SU5402-treated cells had 223 genes that were higher (fold change of 1.4-9.8) and 198 genes that were lower (fold change of 1.4-9.7) in SU5402-treated cells than FGF-2-starved controls. On the other hand, only 30 genes were higher (fold change of 1.4-2.8) and 96 genes lower (fold change of 1.4-3.0) in FGF-2-treated cells than FGF-2-starved controls. Three of the genes that were upregulated in SU5402-treated cells were *ANXA-1-4* (annexins 1-4) and *KRT-8* and -*18* (cytokeratins 8 and 18). The genes that were downregulated in SU5402-treated cells include *POU5F1* (*OCT4*), *DNMT3B*, *TERF1*, and *GAL*. FGF-2-treated hESCs upregulated *LEFTY-1, CASP3, TIMP4, PVRL-3, HESX-1*, and *GAL* genes and downregulated of *SLC16A3*, *TXNIP*, *SRC*, and *PTHR1*. Lists of genes that were regulated by exogenous FGF-2 or SU5402 exposure are shown in supporting information Table 2.

Our analysis of transcriptional responses of hESCs to SU5402 or FGF-2 treatment was performed on selected transcription factor networks and molecular and biochemical pathways. This functional clustering of differentially expressed genes showed an involvement of FGF-2 signaling in cell adhesion, adherens junctions, and transcriptional regulation of genes involved in the self-renewal and differentiation of hESCs. Intrinsic and extrinsic FGF signals had more significant effects on transcriptional regulators than other molecular or biochemical pathways. Functional clustering of deregulated genes is shown in Tables 1 and 2 and supporting information Tables 3 and 4. Taken together, these results show that endogenous autocrine/paracrine FGF signaling plays a crucial role in maintenance of the undifferentiated growth of hESCs.

**Table 1:** Functional clustering of transcription factor regulations associated genes differentially expressed in human ESCs treated with SU5402 or exogenous FGF-2

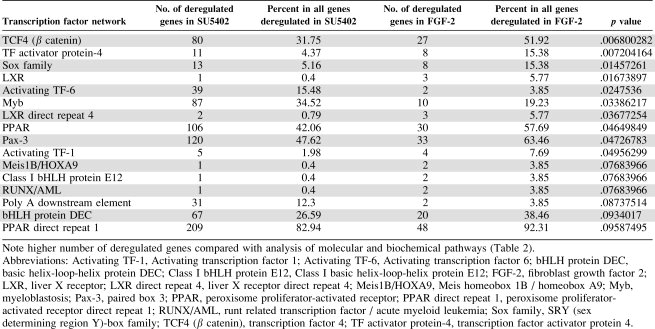

**Table 2:** Functional clustering of molecular or biochemical pathways associated genes differentially expressed in human ESCs treated with SU5402 or exogenous FGF-2

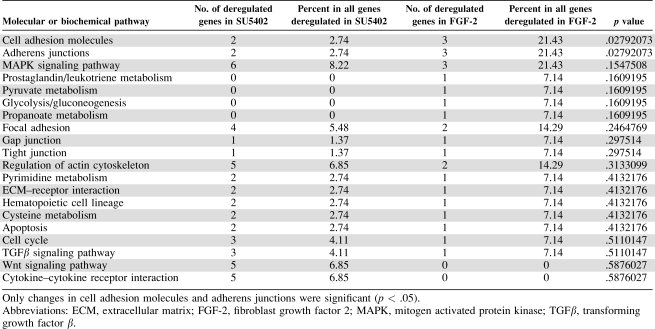

### Expression of Endogenous FGF-2 in hESCs Is Downregulated During Differentiation and Correlates with Increased Binding Capacity for Exogenous FGF-2

**Figure 1:**
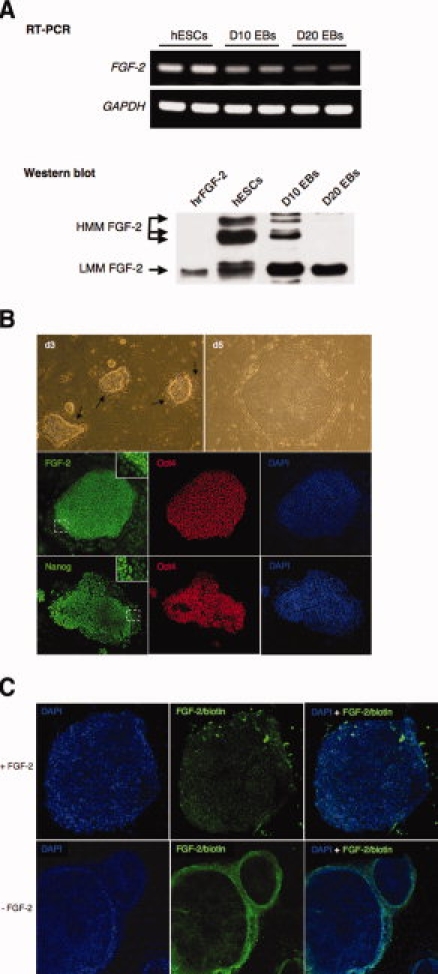
Regulated expression of endogenous FGF-2 correlates with differentiation of hESCs and their binding capacity to exogenous FGF-2. **(A):** RT-PCR of *FGF-2* gene and immunoblotting of 18-, 22-, 22.5-, and 24-kDa FGF-2 isoforms in undifferentiated and differentiating hESCs. **(B):** Peripheral differentiation after FGF-2 withdrawal (arrows) and downregulated nuclear FGF-2, Oct4, and Nanog immunofluorescence. **(C):** Biotinylated FGF-2 (green) binding to undifferentiated hESCs (+FGF-2) or peripherally differentiated hESCs (−FGF-2). Cells are counterstained with 4′,6-diamidino-2-phenylindole (blue). Representative results of triplicate experiments. Abbreviations: EB, embryoid body; FGF-2, fibroblast growth factor-2; GADPH, glyceraldehyde 3-phosphate dehydrogenase; hESCs, human ESCs; RT-PCR, reverse transcriptase-polymerase chain reaction.

Next we wanted to determine whether the expression of endogenous FGF-2 is restricted to undifferentiated hESCs. Endogenous FGF-2 is gradually downregulated during formation and growth of embryoid bodies (EBs). Importantly, this downregulation of FGF-2 is isoform selective. Undifferentiated hESCs expressed nuclear high molecular mass (HMM: 22, 22.5, and 24 kDa) and cytoplasmic low molecular mass (LMM: 18 kDa) isoforms, whereas day 20 EBs expressed only the LMM isoform (Fig. 1A). Correspondingly, when hESC cultures were stimulated to differentiate by withdrawal of exogenous FGF-2 for a shorter period of time (3-5 days), the nuclear FGF-2 was downregulated. This downregulation of endogenous FGF-2 was observed exclusively in a loosely aggregated peripheral hESC subfraction that surrounded the compact central regions of colonies and was accompanied by downregulation of Oct4 and Nanog (Fig. 1B). Proliferation of these peripheral cells appeared to be unaffected (supporting information Fig. 1). It has been reported that these peripheral cells, defined as Oct4/IGF1R-negative fibroblast-like cells, lack clonogenic capacity and upregulate FGFRs that are almost undetectable in Oct4/IGF1R-positive self-renewing hESCs [17]. This suggests that an autocrine/paracrine FGF signaling loop either does not exist or has limited biological significance in a fraction of self-renewing pluripotent hESCs. In contrast, our previous data showed that inhibition of autocrine/paracrine FGF signals causes rapid differentiation, specifically in the central regions of colonies [1]. Moreover, here we show that sorted SSEA3-positive hESCs that possess high clonogenic capacity [24,27,28] express all four FGFRs (supporting information Table 5). To address these contradictions, we performed a series of FGF-2 binding assays with hESCs that were maintained in standard conditions with FGF-2 or cultured without FGF-2 for 3-5 days to induce formation of peripheral fibroblast-like cells. We observed homogenous binding of FGF-2 to hESCs cultured under standard conditions (Fig. 1C, top). However, when hESCs were maintained without exogenous FGF-2 and we observed changes in the morphology of peripheral cells, these cells showed increased FGF-2 binding capacity (Fig. 1D, bottom). This suggests that the controversy in the expression of FGFRs described above may reflect an assay sensitivity issue. Importantly, increased FGF-2 binding capacity closely correlated with downregulation of endogenous FGF-2, Nanog, and Oct4 and likely represents a differentiation phenotype (Fig. 1B). Moreover, a correlation between peripheral differentiation induced by withdrawal of FGF-2 and downregulation of endogenous FGF-2 expression was observed almost exclusively in low passage number hESCs (<50; supporting information Fig. 2), suggesting that FGF-2 requirements change during prolonged cultivation. These results implicate direct intrinsic FGF-2 signaling in the self-renewal of pluripotent hESCs, although an indirect role of exogenous FGF-2 in the creation of an hESC supportive niche by stimulation of hESC-derived peripheral fibroblast-like cells is also likely.

### FGF-2 Knockdown Induces Differentiation of hESCs

**Figure 2:**
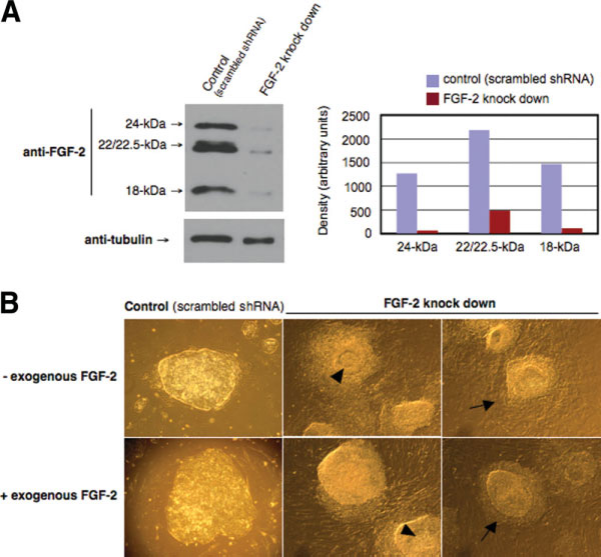
shRNA-mediated knockdown of FGF-2 in human ESCs (hESCs). **(A):** Immunoblot of 18-, 22-, 22.5-, and 24-kDa isoforms of FGF-2. **(B):** hESC morphology after FGF-2 depletion; arrows mark differentiation. Undifferentiated morphology of hESCs was not rescued by addition of 10 ng/ml FGF-2 (+exogenous FGF-2). Representative results of duplicate experiments. Abbreviations: FGF-2, fibroblast growth factor-2; shRNA, small hairpin RNA.

To determine whether endogenously synthesized FGF-2 maintains the undifferentiated growth of hESCs, we transfected hESCs with shRNA directed against all molecular isoforms of FGF-2 and assessed the effects on hESC differentiation. As determined by immunoblot analysis, this approach reduced the expression of the 18- and 24-kDa isoforms of FGF-2 by approximately 90% and the 22/22.5-kDa isoforms by 70% (Fig. 2A). Transfection of hESCs with scrambled shRNA had no effect on the expression of endogenous FGF-2. In hESCs transfected with the FGF-2 specific shRNA, but not in cells transfected with control shRNA, we consistently observed increased differentiation evidenced by flattened cells in the centers of hESC colonies and peripheral fibroblast-like cells (Fig. 2B). This effect was visible when hESCs reached higher densities in approximately 60%–90% of all colonies, suggesting that residual action of the remaining FGF-2 was sufficient to maintain undifferentiated phenotype in some colonies. Importantly, this effect of FGF-2 knockdown on the undifferentiated growth of hESCs was likely not caused by the partial absence of the18-kDa isoform of FGF-2, because differentiation was also observed in hESC cultures supplemented with exogenous 18-kDa FGF-2. These data reinforce the role of intracrine FGF-2 HMM isoforms in maintenance of the undifferentiated growth of hESCs.

### FGF-2 Signals in Undifferentiated hESCs Dominantly via FGFR2 and Activates MAPKs and Akt Kinase

**Figure 3:**
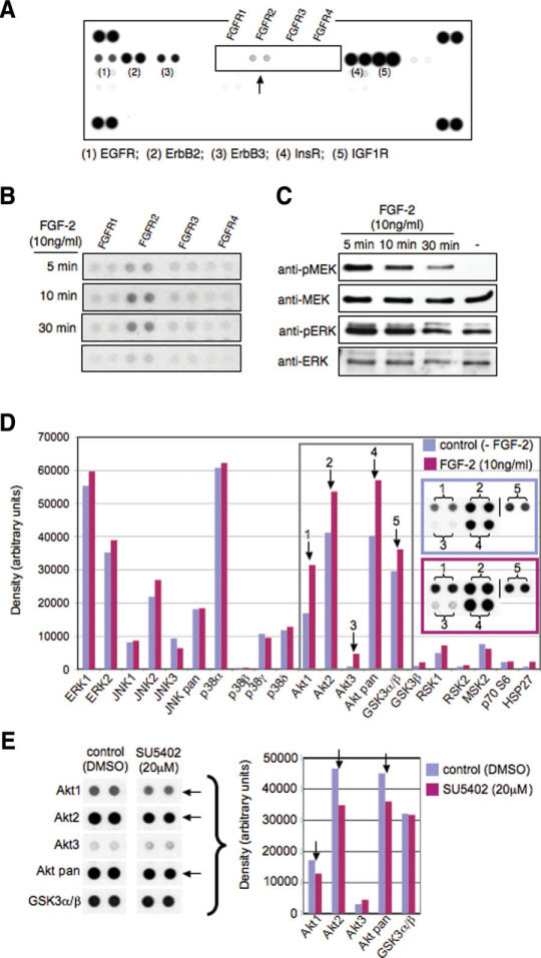
FGF-2 signaling via FGF receptor 2, mitogen-activated protein kinases, and Akt. **(A):** Phospho-receptor kinases protein array of FGF-2 starved human ESCs (hESCs) or **(B)** after FGF-2 treatment. **(C):** Immunoblot analysis of MEK and ERK in time course FGF-2-treated hESCs. MAPK protein array of hESCs treated with **(D)** FGF-2, **(E)** SU5402, or vehicle (DMSO). Representative results of more than five experiments are shown. Abbreviations: DMSO, dimethylsulfoxide; EGFR, epidermal growth factor receptor; ERK, extracellular signal-regulated kinase; FGF-2, fibroblast growth factor-2; IGF1R, insulin-like growth factor 1 receptor; MEK, mitogen-activated protein kinase kinase.

Undifferentiated hESCs synthesize and release LMM FGF-2 and express all four FGFRs (Fig. 1) [1]. Thus, FGF-2 autocrine/paracrine signaling can take place. To assess the function of FGF-2 autocrine/paracrine signaling, we performed phospho-receptor kinase protein arrays in samples from hESCs maintained without human recombinant FGF-2 for 4 days. The dominantly phosphorylated active high affinity FGF receptor was FGFR2 (Fig. 3A) in exogenous FGF-2-starved samples, which is a relatively weakly transcribed FGFR in hESCs (supporting information Table 5) [1]. In agreement with recently published data [20], the most phosphorylated receptor tyrosine kinases were IGF1R, insulin receptor, and members of the EGFR family (EGFR, ErbB2, and ErbB3; Fig. 3A). Tyrosine phosphorylation of the FGFRs starts immediately after ligand-receptor binding with kinetics that determine subsequent receptor isoform-specific activation and recruitment of intracellular signaling molecules. Thus, we subjected FGF-2-starved hESCs to 5-, 10-, and 30-minute stimulation with exogenous FGF-2, which showed that phosphorylation of all four FGFRs increased with a maximal response of FGFR2 10 minutes after the addition of FGF-2 (Fig. 3B). Downstream of FGFRs, activated FRS2/Grb2 complexes recruit SOS or GAB1 and activate the Ras-Raf-MAPK signaling cascade or the PI3K-Akt cell survival pathway, respectively. The MAPK and PI3K-Akt pathways can also be stimulated by activated EGF family receptors, insulin receptors, and IGF1Rs (Fig. 3A). Thus, activation of FGFRs may be hidden and may produce only weak cumulative effect on these pathways. To address this question, we examined the kinetics of FGF-2-mediated phosphorylation of MEKs and ERKs. Despite the high levels of basal ERK activation, the phosphorylation of both MEK and ERK significantly increased, with a maximal response 5 minutes after the addition of FGF-2 (Fig. 3C). Next, we examined the effects of FGF-2 on a larger panel of MAPKs and the Akt kinase family using a proteome profiler phospho-MAPK array. A 5-minute treatment with FGF-2 caused activating phosphorylation of ERK, Akt, and GSK3α/β (Fig. 3D). The phosphorylation of Akt kinases was 30% higher than controls, whereas ERK1 and 2 increased only 8%–10%. These data suggest that exogenous FGF-2 primarily affects the balance between survival and apoptosis in hESCs. To test this hypothesis for endogenously produced FGF-2, we inhibited FGF autocrine/paracrine signaling with SU5402, treated hESCs with human recombinant FGF-2 in parallel, and again analyzed the phosphorylation level of kinases using phospho-MAPK arrays. Akt1, Akt2, and pan-Akt phosphorylation levels were lower in cells exposed to the SU5402 FGFR inhibitor compared with cells that were starved of exogenous FGF-2 (Fig. 3E), whereas exogenous FGF-2 had the same activating effect as shown in Figure 3D. Our results suggest that both exogenous and endogenous FGF-2 regulate Akt kinase signaling.

### The 18-kDa Isoform of FGF-2 Regulates hESC Survival

**Figure 4:**
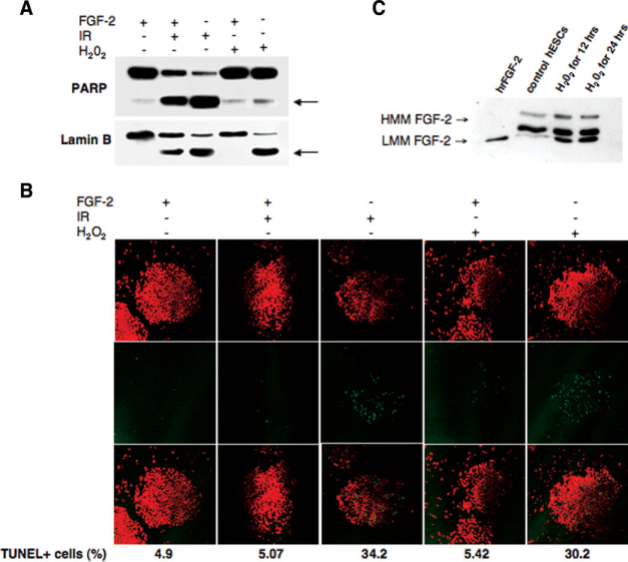
Human ESC (hESC) apoptosis and increased expression of FGF-2 in response to cellular stress. **(A):** Immunoblot of PARP and lamin B cleavage (3 hours after treatment), and **(B)** quantitative TUNEL (12 hours after treatment) of hESCs FGF-2 prestarved (6 hours) and incubated with 10 ng/ml FGF-2 (2 hours) and irradiated (1 Gy) or exposed to H_2_O_2_ (50 μM, 1 hour). Control cells were irradiated or exposed to H_2_O_2_ in the absence of exogenous FGF-2. **(C):** hESCs were treated with 25 μM H_2_O_2_ without exogenous FGF-2 for 12 and 24 hours and immunoblotted for endogenous FGF-2. Control cells were maintained without H_2_O_2_. Representative results of triplicate experiments. Abbreviations: FGF-2, fibroblast growth factor-2; IR, irradiated; PARP, poly(ADP-ribose) polymerase; TUNEL, terminal deoxynucleotidyl transferase-mediated dUTP nick end labeling.

To examine whether FGF-2 protects hESCs from apoptosis induced by cellular stress, FGF-2-treated and untreated hESCs were subjected to ionizing radiation or oxidative stress and cell death was assessed by lamin B and PARP cleavage, and terminal deoxynucleotidyl transferase-mediated dUTP nick end labeling assays. Human ESCs grown in the presence of exogenous LMM FGF-2 were protected from cell death induced by cellular stress (Fig. 4A, 4B). Also, oxidative stress consistently increased the expression of LMM FGF-2 by 12 and 24 hours after treatment, suggesting that hESCs synthesize FGF-2 to in response to cellular stress (Fig. 4C). In addition, high concentrations of FGF-2 (100 ng/ml) in hESC cryopreservation medium enhances the post-thaw viability of hESCs by approximately 15% and significantly increases the number of attached recovered cells (supporting information Fig. 3A, 3B). Collectively, these results support the hypothesis that LMM FGF-2 supports cell survival.

### Exogenous FGF-2 Supports the Cloning Efficiency of hESCs by Enhancing Cellular Adhesion and Survival

**Figure 5:**
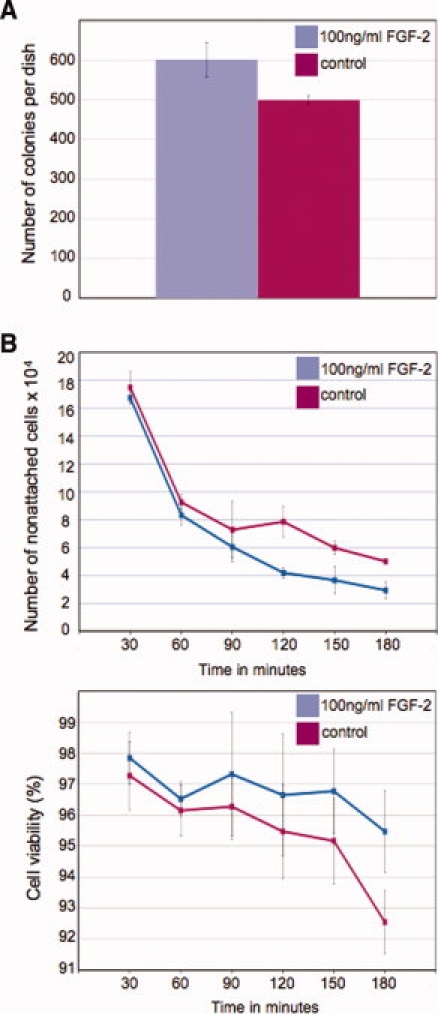
FGF-2 affects the cloning efficiency of human ESC (hESC) single cell suspensions. hESCs were preincubated and seeded in medium with FGF-2 (100 ng/ml) or without FGF-2. **(A):** Number of alkaline phosphatase-positive colonies; numbers represent the mean of 24 wells of one 24-well plate. **(B):** Number and viability of nonattached hESCs. Representative results of more than five experiments are shown. Abbreviations: FGF-2, fibroblast growth factor-2.

The lower survival rates of hESCs that have been dissociated into single cell suspensions are likely to be the result of cellular stress for a cell type that normally grows in tightly packed colonies within a tightly regulated stem cell niche. To test this hypothesis, cells were completely dissociated with TrypLE reagent, preincubated in suspension with high concentrations of FGF-2 (50-100 ng/ml) for 2 hours, and seeded into feeder cell-coated dishes in medium with the same concentrations of FGF-2. After overnight culture, medium with high concentrations of FGF-2 was changed to a standard medium with 5 ng/ml FGF-2, and cells were cultured for 3-4 days and stained for alkaline phosphatase. As shown in Figure 5A, the number of recovered colonies (cloning efficiency) was higher when hESCs were preincubated and seeded with FGF-2. However, the cloning efficiency with or without FGF-2 was not significantly different when we combined stress from single cell dissociation with oxidative stress (data not shown). This raises the possibility that increased cloning efficiency might also reflect enhanced cell attachment in cells that were preincubated with FGF-2. Therefore, we designed a cell attachment assay where we counted the numbers of and assessed the viability of nonattached hESCs plated either with or without FGF-2. The numbers of nonattached cells were consistently higher, and viability was consistently lower in hESC cultures that were incubated without exogenous FGF-2 (Fig. 5B). These results show that the stimulatory effect of FGF-2 on cloning efficiency results from a combined enhancement of cell survival and adhesion.

## DISCUSSION

Our experiments have for the first time identified FGF-2 as an intrinsic regulator of hESC self-renewal and survival. Our results also suggest that the human recombinant FGF-2 added to hESC culture media promotes cell adhesion and survival, but its effect on the maintenance of pluripotency gene expression is minor. We postulate that exogenous FGF-2 is required for the undifferentiated growth of hESCs because it promotes cell adhesion, and thus, growth of compact and regularly shaped colonies that are less prone to peripheral differentiation. Our observation that exogenous FGF-2 increases replating efficiency of hESCs contradicts an earlier study [29], in which authors did not observe significant improvement in the replating efficiency in the presence of increased concentrations of FGF-2. However, we used TrypLE reagent rather than trypsin/EDTA, single cell suspensions were immediately transferred into FGF-2 high culture medium, and cells were preincubated before seeding. Given that FGF-2 can rescue cells exposed to cellular stress ([30] and our results) and stimulate cell adhesion, these differences in our experimental design may have enhanced the survival and adhesion of the single cell suspensions. Direct effects of exogenous FGF-2 on hESC adhesion and survival that were uncovered by our study may be similarly important as the action of exogenous FGF-2 on feeder cells to stimulate TGFβ1 and activin A signaling [15,16]. In addition, endogenous production of FGF-2 by undifferentiated hESCs supports an intrinsic pluripotency maintenance program. This second pathway raises several questions. Is the intrinsic FGF-2 pathway a continuation of the developmentally predetermined self-renewing program of epiblast cells or is it an artifact acquired in vitro that ensures the survival and undifferentiated proliferation of hESCs? Is the activity of FGF-2 critical for hESC adaptation to in vitro culture conditions and, to a certain extent, similar to the transforming activity of CUG-initiated HMM isoform of FGF-2 in other cell types [31]? Do the high and low molecular mass isoforms of endogenously synthesized FGF-2 have such distinct functions as described in other cell types [32,33]? To partially answer these questions, FGF-2 is known to affect uterine receptivity [34–36] but was not detected in mammalian blastocyst stage embryos. This suggests that the expression of FGF-2 in hESCs is an acquired adaptation to in vitro culture conditions. Similar adaptation to culture conditions has been observed for chondrocytes isolated from human fetal growth plate cartilage, which exhibits culture-induced expression of FGF-2 HMM isoforms [37], and several mouse-derived cell types cultured from tissue biopsies (P. Krejci, personal communication).

Whether or not FGF-2 requirements are an in vitro adaptation, endogenous FGF-2 clearly plays a critical role in the undifferentiated growth of hESC in culture. The expression of HMM FGF-2 has also been shown to enhance proliferation and survival in suboptimal culture conditions for various cell types, and it also stimulates cellular transformation and stress/drug resistance [22]. Our observation that nuclearly localized HMM FGF-2 is rapidly downregulated when hESCs start to differentiate at the periphery of tightly packed colonies as a consequence of withdrawal of exogenous FGF-2 suggests that LMM FGF-2 participates in the creation of a stem cell niche by supporting cell adhesion. It also suggests that endogenously produced FGF-2, particularly the HMM isoforms, decides hESC fate. LMM FGF-2 is a well-established autocrine/paracrine regulator of normal fibroblast proliferation [38] and invasiveness and tumorigenic potential of carcinoma cells [39]. Similar mechanisms may underlie the ability of endogenously produced FGF-2 to facilitate the adaptation and growth of inner cell mass (ICM)-derived cells in vitro. The intrinsic activities of FGF-2 may also be complemented by the intrinsic activities of FGF-4, another FGF family member that was recently suggested to be a regulator of hESC proliferation and differentiation [40].

Our results also showed that FGFR2 is the key signaling receptor for exogenous FGF-2 in hESCs, even though its expression is much lower than another FGF-2-cognate receptor, FGFR1. This is consistent with the role of FGFR2 during mammalian development, where FGFR2 regulates the outgrowth and maintenance of the ICM, basement membrane formation, and epithelial differentiation [41–44]. Simultaneously, this raises the issue of what the role of dominantly expressed FGFR1 is in hESCs. FGFR1 may support the attachment of hESCs by forming nonsignaling complexes with FGF-2 and the feeder cell-associated extracellular matrix, heparin sulfate proteoglycans, or cell adhesion molecules as it does in myeloid and neural cells [45,46]. Future studies are underway to determine whether FGFR1 knockdown affects hESC adhesion, differentiation, or MAPK activation.

## CONCLUSION

In conclusion, FGF-2 plays a complex multilevel role in the maintenance of hESC pluripotency. Although intracrine FGF-2 signaling maintains pluripotency gene expression, the exogenous recombinant hFGF-2 supplementation of hESC culture media primarily contributes to the maintenance of hESC pluripotency by promoting cell adhesion and survival. This complex mechanism of action may also apply to other growth factor regulators of hESC pluripotency in vitro and represents a new approach to the research of the maintenance of the undifferentiated growth of hESCs.
