# Supplementary figures and images for: A Complex Role for FGF-2 in Self-Renewal, Survival, and Adhesion of Human Embryonic Stem Cells
Source: Stem Cells. 2009 Aug;27(8):1847–57. doi: 10.1002/stem.128 (PMC2798073; doi:10.1002/stem.128)

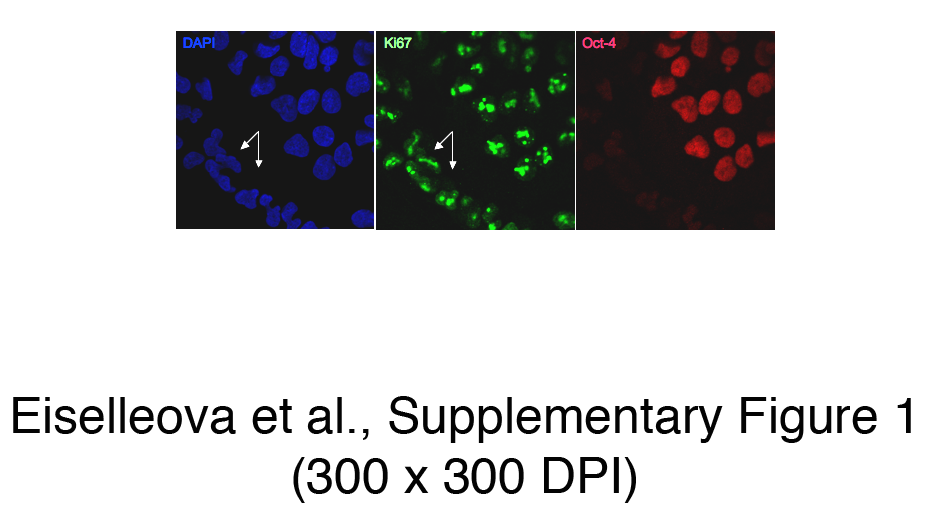

Supplement: Supplementary file 1 [file stem0027-1847-SD1.tif]

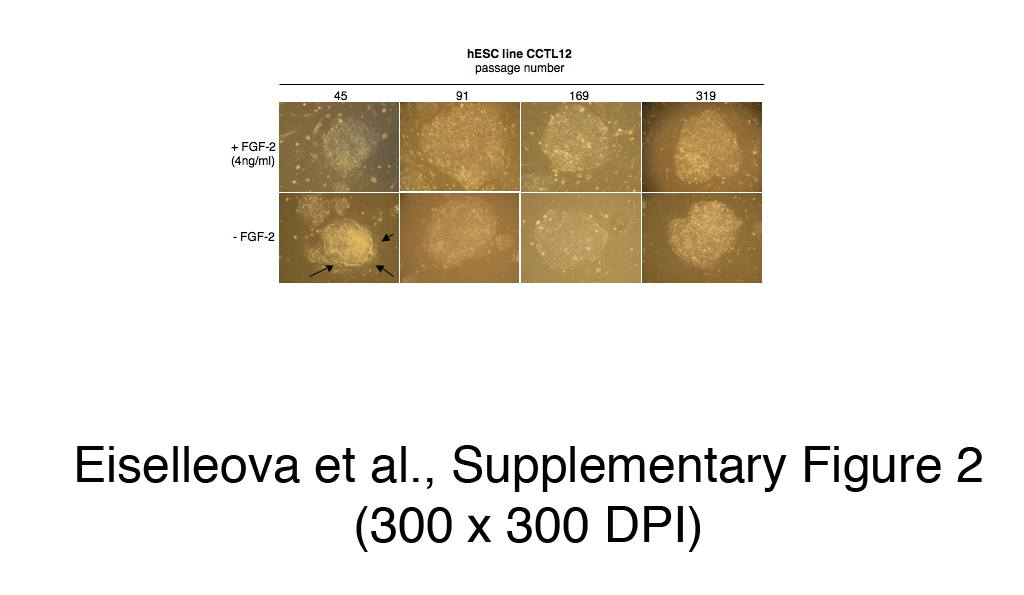

Supplement: Supplementary file 2 [file stem0027-1847-SD2.tif]

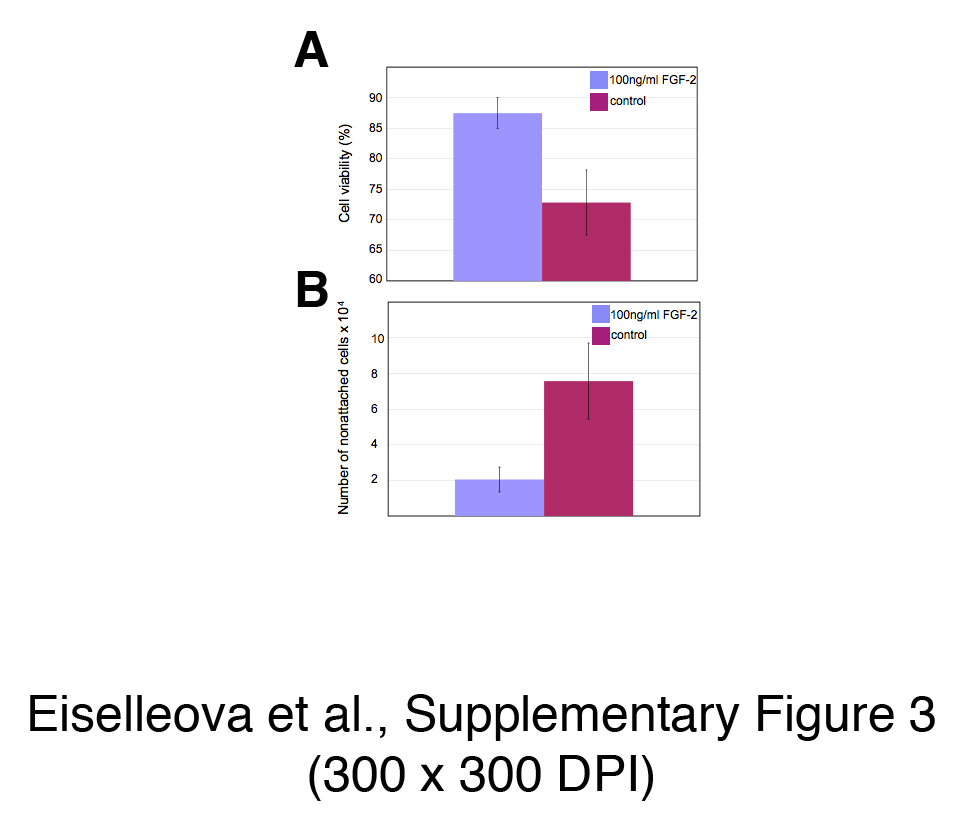

Supplement: Supplementary file 3 [file stem0027-1847-SD3.tif]
