# Supplementary material for: A Complex Role for FGF-2 in Self-Renewal, Survival, and Adhesion of Human Embryonic Stem Cells
Source: Stem Cells. 2009 Aug;27(8):1847–57. doi: 10.1002/stem.128 (PMC2798073; doi:10.1002/stem.128)
Supplement: Supplementary file 4 [file stem0027-1847-SD4.doc]

Supplementary Table 1 (Eiselleova et al.)

**Gene Sequence of primers Product size (bp)**

**RT-PCR** FGF-2 F: 5‘-GCAGAAGAGAGAGGAGTTGTGTC-3’ 202

R: 5’-ACTGCCCAGTTCGTTTCAGT-3’

GAPDH F: 5‘-AGCCACATCGCTCAGACACC-3’ 302

R: 5’-GTACTCAGCGCCAGCATCG-3’

**Sequence of primers* and probes****

**qRT-PCR** FGFR1 F: 5’-GGACTCTCCCATCACTCTGCAT-3’ 109

R: 5’-CCCCTGTGCAATAGATGATGATC-3’

Probe: 5’-TCATCACTGCCGGCCTCTCTTCCA-3

FGFR2 F: 5’-ACGTGGAAAAGAACGGCAGTA-3’ 101

R: 5’-AGCCAGCACTTCTGCATTGG-3’

Probe: 5’-ACGGGCTGCCCTACCTCAAGG-3’

FGFR3 F: 5’-AGGATGCCTGCATACACACTGC-3’ 224

R: 5’-ACACCCTACGTTACCGTGCTCAAG-3’

Probe: 5’-ACACCTGCCTGGCGGGCAATTC-3’

FGFR4 F: 5’-GAACCGCATTGGAGGCATT-3’ 102

R: 5’-TTCTCTACCAGGCAGGTGTATGTG-3’

Probe: 5’-TCCATCACGAGACTCCAGTGCTG-3’

*The primer pairs for qRT-PCR were located in two separate exons and were confirmed not to produce amplification artifacts.

**TaqMan probes were labeled by a 5’FAM reporter and 3’BHQ1 quencher.

As a control gene for qRT-PCR we used Abelson (ABL). The primers and probe for the ABL gene were designed as described

in Dvorak et al. [1]. Abbreviations: bp, base pairs; F, forward; R, reverse; GAPDH, glyceraldehyde-3-phosphate dehydrogenase
