# Supplementary material for: A Complex Role for FGF-2 in Self-Renewal, Survival, and Adhesion of Human Embryonic Stem Cells
Source: Stem Cells. 2009 Aug;27(8):1847–57. doi: 10.1002/stem.128 (PMC2798073; doi:10.1002/stem.128)
Supplement: Supplementary file 8 [file stem0027-1847-SD8.doc]

Supplementary Table 5 (Eiselleova et al.)

**PASSAGE No. FGFR1 FGFR2 FGFR3 FGFR4**

**Unfractionized cells**

CCTL14 45 406.49 15.73 18.52 45.94

CCTL14 114 288.78 17.88 20.55 93.26

HS237 21 734.75 43.34 62.46 51.28

HS237 94 265.80 17.19 15.23 43.75

**Sorted SSEA3-positive cells**

H7 S14 59 208.25 4.48 11.93 25.15

H7 S6 120 235.65 3.96 21.33 31.89
